# Nanomaterial Lipid-Based Carrier for Non-Invasive Capsaicin Delivery; Manufacturing Scale-Up and Human Irritation Assessment

**DOI:** 10.3390/molecules25235575

**Published:** 2020-11-27

**Authors:** Phunsuk Anantaworasakul, Songyot Anuchapreeda, Songwut Yotsawimonwat, Ornchuma Naksuriya, Suree Lekawanvijit, Napatra Tovanabutra, Pimporn Anantaworasakul, Wajee Wattanasri, Narinthorn Buranapreecha, Chadarat Ampasavate

**Affiliations:** 1 Department of Pharmaceutical Sciences, Faculty of Pharmacy, Chiang Mai University, Chiang Mai 50200, Thailand; phunsuk@gmail.com (P.A.); songwut.y@cmu.ac.th (S.Y.); ornchuma.n@cmu.ac.th (O.N.); 2 Division of Clinical Microscopy, Department of Medical Technology, Faculty of Associated Medical Sciences, Chiang Mai University, Chiang Mai 50200, Thailand; songyot.anuch@cmu.ac.th; 3 Center for Research and Development of Natural Products for Health, Chiang Mai University, Chiang Mai 50200, Thailand; 4 Department of Pathology, Faculty of Medicine, Chiang Mai University, Chiang Mai 50200, Thailand; suree.lek@cmu.ac.th; 5 Division of Dermatology, Department of Internal Medicine, Faculty of Medicine, Chiang Mai University, Chiang Mai 50200, Thailand; napatra.to@cmu.ac.th; 6 International Research and Academic Service Center, International College of Digital Innovation, Chiang Mai University, Chiang Mai 50200, Thailand; pimporn.a@cmu.ac.th; 7 BLC Research Center, Bangkok Lab & Cosmetic Co., Ltd., Ratchaburi 70000, Thailand; wajee@bangkoklab.co.th (W.W.); narinthorn@bangkoklab.co.th (N.B.)

**Keywords:** capsaicin, chili extract, NLC, topical delivery system, human skin irritation

## Abstract

Capsaicin is an active compound in chili peppers (*Capsicum chinense*) that has been approved for chronic pain treatment. The topical application of high-strength capsaicin has been proven to reduce pain; however, skin irritation is a major drawback. The aim of this study was to investigate an appropriate and scalable technique for preparing nanostructured lipid carriers (NLCs) containing 0.25% capsaicin from capsicum oleoresin (NLC_C) and to evaluate the irritation of human skin by chili-extract-loaded NLCs incorporated in a gel formulation (Gel NLC_C). High-shear homogenization with high intensity (10,000 rpm) was selected to create uniform nanoparticles with a size range from 106 to 156 nm. Both the NLC_C and Gel NLC_C formulations expressed greater physical and chemical stabilities than the free chili formulation. Release and porcine biopsy studies revealed the sustained drug release and significant permeation of the NLCs through the outer skin layer, distributing in the dermis better than the free compounds. Finally, the alleviation of irritation and the decrease in uncomfortable feelings following the application of the Gel NLC_C formulation were compared to the effects from a chili gel and a commercial product in thirty healthy volunteers. The chili-extract-loaded NLCs were shown to be applicable for the transdermal delivery of capsaicin whilst minimizing skin irritation, the major noncompliance cause of patients.

## 1. Introduction

The transdermal route is one way of administering active compounds on the skin to overcome limitations such as first pass metabolism or target delivery to a specific skin layer [1]. Lipid-based nanocarriers in the form of nanostructure lipid carriers (NLCs) have gained much attention as an alternative to solid lipid nanoparticles (SLNs) for drug delivery, to overcome the limitations of SLNs such as drug leakage during storage and low drug-loading capacity [2,3]. These carriers are advantageous for several routes of administration: the parenteral, oral, especially topical routes [4,5]. For safety reasons, most of the lipids used in the lipid matrices for SLN and NLC fabrication are physiological and biodegradable components with GRAS (generally recognized as safe) status and are frequently used in commercially available topical cosmetic or pharmaceutical preparations [4,6]. The small size lipid nanoparticles exhibit an occlusive effect that improves skin hydration and increases drug permeation [7]. Further advantages of their use for administering pharmaceuticals include their ability to control a drug’s release, reduce its side effects, and target its delivery [8].

Capsaicinoids, major active compounds in chilis (*Capsicum chinense*), have been used in folk medicine for their therapeutic effects, including analgesic and anti-inflammatory effects, and inhibition of gastrointestinal, cardiovascular, and respiratory diseases [9]. Capsaicin (8-methyl-*N*-vanillyl-trans-6-nonenamide) is an active compound and is used as a marker for chili extracts. The benefits of capsaicin in topical applications for various diseases such as musculoskeletal pain, osteoarthritis, rheumatoid arthritis, diabetic neuropathy, and post-herpetic neuralgia have been proven [10,11]. Commercial products are available in the forms of creams, gels, lotions, ointments, or patch formulations—typically containing 0.0125–0.075% capsaicin by weight—for chronic pain management, with application three to five times daily for a few months [12]. The pharmacological mechanisms of capsaicin in pain control are associated with TRPV1 receptors (transient receptor potential vanilloid subfamily, member 1), expressed on nociceptive fibers in the dermis. However, the important problem with chili extract after its application on the skin is severe irritation when capsaicin binds the TRPV1 receptors presented on the keratinocytes in the epidermis [9]. The unwanted side effects of capsaicin products include a burning sensation, leading to severe skin irritation and poor patient compliance [9,11]. The development of lipid nanocarriers has been suggested in order to harness the benefits of capsaicin while avoiding its irritative effects.

Previous studies have reported several techniques for preparing chili-extract-loaded lipid-based nanocarriers such as high-pressure homogenization (HPH) [13,14,15], high-shear homogenization (HSH) [13,14,16], microemulsion techniques [17,18,19], emulsification sonication [8,16], solvent-emulsification evaporation [19,20], and solvent-diffusion methods [21]. In the solvent-emulsification evaporation and solvent-diffusion techniques, hazardous organic solvents such as chloroform and dichloromethane were required for the preparation, as well as the emulsification sonication technique in which metal contamination from a sonicating probe was observed in the products [22,23]. These are the limitations of most techniques which were developed for lab-scale production. The potential of high-pressure homogenization and the microemulsion method for manufacturing scale-up has been demonstrated [24,25]. The standard method for NLC fabrication using combination technologies consists of high-shear or high-speed homogenization employed in the pre-emulsion step followed by HPH or emulsification sonication [23,25,26]. However, these techniques have limitations regarding performance, such as long particle-size-reduction times, low production capacity, complexity of operation, and the clean-up process, especially in large-scale or industrial-scale production [27]. To avoid multiple steps in nanoparticle production, the HSH—a technique typically used in the pre-emulsion step—can be developed as a single apparatus for lipid-nanoparticle production by the optimization of factors such as the design and size of the generator, speed rate, processing time, viscosity of the medium, and volume of the sample [28].

The main purpose of this study was to investigate a suitable technique for preparing chili-extract-loaded NLCs containing a high concentration of capsaicin (0.25%) from chili oleoresin extract to enhance the transdermal delivery and the sustained release of capsaicin, while reducing skin irritation. The physicochemical characteristics of the particles obtained from three scale-up techniques, such as the physical and chemical stabilities, were compared to identify the most optimal technique for industrial-scale production. The developed chili-extract-loaded NLCs were incorporated in a gel formulation containing 0.075% capsaicin. The irritancy of the final formulae was evaluated in humans.

## 2. Results and Discussion

### 2.1. Characterization of NLCs Prepared by Different Methods

**Figure 1:**
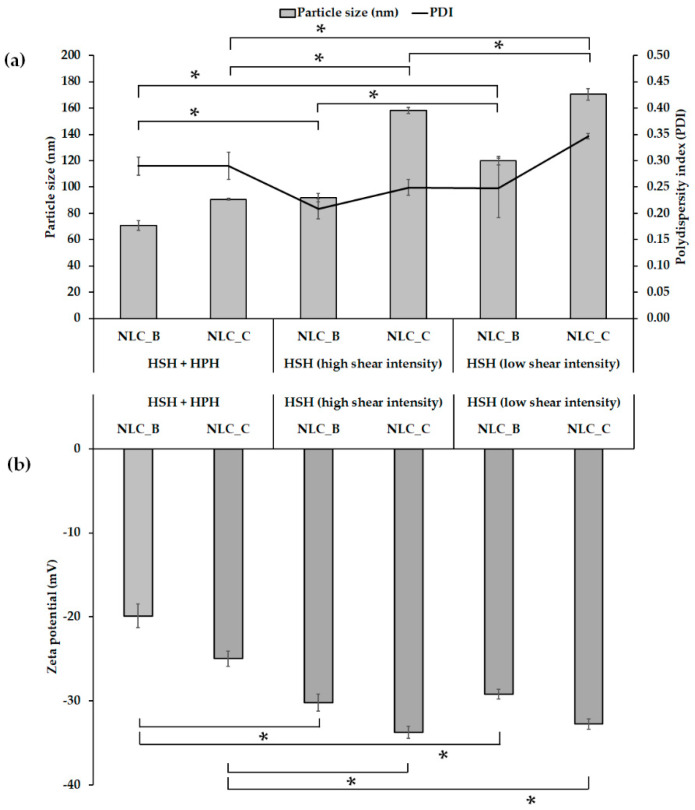
Influence of techniques for nanostructured lipid carriers (NLC) fabrication on (**a**) the particle size and size distribution (PDI) and (**b**) zeta potential. The techniques included (1) a combination of high-shear homogenization (HSH) and high-pressure homogenization (HPH), (2) HSH with a high shear intensity, and (3) HSH with a low shear intensity. Abbreviations: NLC_B, blank NLC; NLC_C, chili-extract-loaded NLC. * The particle size and zeta potential values are significantly different (*p* < 0.05) between the methods.

Many previous studies had confirmed the superior entrapment efficiency of NLCs compared to that of SLN [2,29,30]. Chili-extract-loaded NLCs (NLC_Cs) were prepared from a lipid mixture and surfactant as described in the previous study [29]. Besides the chemical composition used in the formulations, the production method is a key factor contributing to the characteristics of the lipid nanoparticles. The widely used SLN or NLC production techniques include, but are not limited to, HPH, HSH, ultrasonication, microemulsion, and solvent-emulsification-evaporation techniques [23,26]. Organic solvents are normally used in the solvent-emulsification-evaporation or solvent-dispersion techniques. The evaporation or disposure of the organic solvents creates complexity for the scaling-up process [22,23]. To limit the complexity of the production and the use of hazardous organic solvents, this study employed the HPH and/or HSH methods for NLC production. Both methods produced small-size particles, smaller than 200 nm. As shown in Figure 1a and Table 1, the combination of two processes (HSH and HPH) produced the smallest particle size, followed by NLC production with the HSH with a high shear intensity for a short period (10 min) and the HSH with a low shear intensity for a longer period (30 min), respectively. The particle size produced by the HSH+HPH method was significantly smaller than that produced by the other methods (*p* < 0.05), and the high shear intensity significantly reduced the particle size compared to that achieved with the low shear intensity (*p* < 0.05). The size reduction mechanism of the high-pressure homogenizer basically consists of a positive-displacement pump attached to a homogenizing valve assembly. The pump forces the melted lipid mixture fluids through the homogenizing valve under pressure. The HPH can operate on a combination of shear stress and cavitation forces [31,32]. The HSH produces a smaller droplet size using a rotor-stator mixer set based on a stationary stator coupled with an inside rotor responsible for the high speeds [31]. In this study, all the methods provided a narrow particle size distribution in the NLC dispersions, with polydispersity indices (PDI) less than 0.3. The PDI values obtained with all the methods were not significantly different (*p* > 0.05) on the day after preparation (Day 0). Their surfaces were negatively charged, with zeta potentials ranging from −20 to −35 mV. The characteristics of NLC dispersion on the initial day (Day 0) in terms of particle size and PDI are shown in Figure 1a, and the zeta potentials are shown in Figure 1b. 

### 2.2. NLC Stability

The stability of the NLCs, blank NLCs (NLC_Bs), and chili-extract-loaded NLCs (NLC_Cs) produced by the different methods were observed after storage in closed containers at room temperature for 30 days. The particle size of the NLC_Cs significantly increased after 14 days of storage, except for those produced by HSH with a high shear intensity. Table 1 shows the stability trends of the NLCs produced by the different techniques after a month of storage, in terms of the particle size, size distribution, and surface charge. These stability results show the potential of HSH operated at high intensity for preparing NLC_C dispersions. Although HPH is the most commonly used technique for lipid-based nanoparticle fabrication because it can form small particle sizes, it results in coalescence and agglomeration upon storage due to the particles that were separated by the powerful HPH re-combining after the dissipation of the high kinetic energy [26,33]. In addition, the high temperature and shear stress from the process could induce gel formation by increasing the kinetic energy of the particles and their collision; instability of the nanoparticles upon crystallization was induced by a gelling phenomenon [32]. Stability could be achieved by adding sufficient surfactants; however, these are directly related to irritation [32,34]. The factors influencing the stability and size of the particles produced by the HSH method depended on the shear forces and turbulent flow, which produced less energy than those in the HPH method [35]. The HSH is a technique normally used for the coarse-emulsion production (the pre-emulsion step) of lipid-based nanoparticle preparations in the laboratory, pilot, and industrial scales. This technique is easy to handle, cost-effective and less time-consuming, providing nanometric particle sizes along with good formulation development [36,37]. Furthermore, the high-shear homogenizer is a versatile piece of equipment that is widely used as a mixer for preparing various pharmaceutical dosage forms, such as emulsions, creams, lotions, and gels [38]. The parameters influencing the particle size were not only the formulation methods but also the conditions (such as the time and force) in the process, shear intensity, and shear time in the HSH, as well as the pressure and number of homogenization cycles in the HPH. The size distribution and zeta potential are also key factors in the prediction and evaluation of the stability of colloid dispersions. The size distribution affects the physical stability and uniformity of NLCs according to the polydispersity index (PDI) value. The PDI value should be as low as possible for long-term stability. PDI values of 0.1–0.25 show a narrow size distribution, while those greater than 0.5 imply a broad distribution [39]. The PDI values of the NLCs prepared by all three techniques were approximately 0.25–0.3 on the first day after preparation, while by the thirtieth day of storage, the combination technique (HSH + HPH) and HSH with a low shear intensity showed PDI values above 0.5, which could be related to particle growth or flocculation. Apart from the particle size, a zeta potential (ZP) above ±30 mV was only observed from the production with the high shear HSH technique, representing good electrostatic stabilization, where the attractive forces were lower than the repulsion forces [40]. Thus, for a combination of electrostatic and steric stabilization, an absolute zeta potential value of about ±20 mV is advised [39]. In this study, the zeta potential values of all the formulations were between −20 and −40 mV. After thirty days of storage, only the zeta potential of the NLC dispersion from the HSH with high shear intensity showed an absolute value higher than ±20 mV, which is connected with the good stability of the colloidal system. As shown in Table 1, the stability of the NLCs produced from the high shear intensity HSH was higher than that of those from the HSH-followed-by-HPH and low-shear-intensity-HSH techniques. Other techniques, such as melt-dispersion ultrasonication, film ultrasound, and solvent diffusion, were reported to produce broad distributions of particle sizes, with formulations of low predicted physical stability. In addition, their complex processes created some difficulties for NLC large-scale production [41]. A widely used and well-established method for NLC production is high-pressure homogenization (HPH); however, an NLC formulation produced by HSH with a high shear intensity is possible [25,42,43]. 

**Figure 2:**
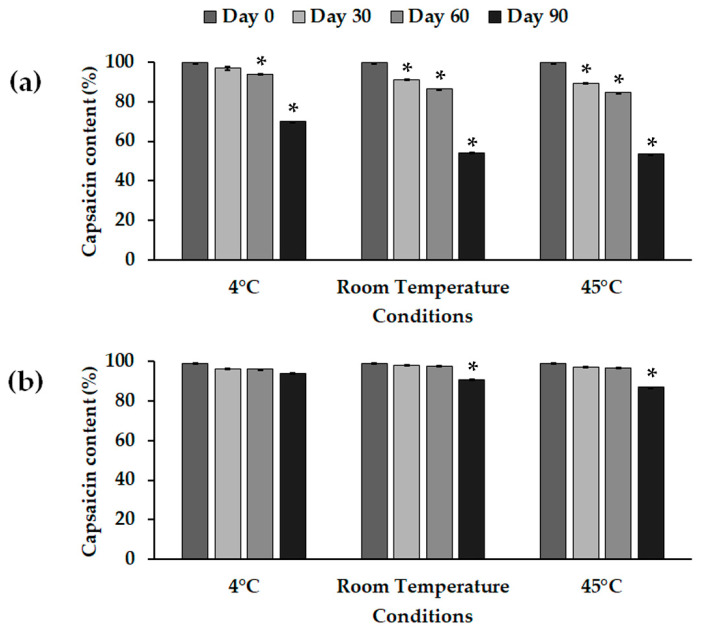
Percentages of capsaicin contents following 90 day storage of (**a**) chili-extract solution and (**b**) NLC_C, prepared by HSH with high shear intensity, at different conditions: 4 °C, room temperature (RT), and 45 °C. * The values are significantly different (*p* < 0.05) compared to those for the initial formulation (Day 0).

According to the particle sizes of the NLCs produced by the different methods, the NLC_Cs produced using HSH with high shear intensity were chosen for the scale-up NLC preparation. A stability study at different temperatures—4 °C, room temperature (RT) and 45 °C—for 90 days was performed. The physical stability of NLC_C showed no significant difference in terms of the particle size, size distribution, zeta potential, and physical characteristics—i.e., phase separation—under 4 °C and RT storage conditions for 90 days, whereas high-temperature (45 °C) storage induced a significant difference in particle size from that at Day 0; the characteristic data are shown in Table 2. Moreover, an enhancement of chemical stability after incorporation into lipid-based nanocarriers was proven in terms of the remaining capsaicin content [42]. In this study, the NLC_C dispersion was stable and more effectively stabilized the capsaicin than the chili-extract solution. The remaining capsaicin contents in the NLC_Cs showed significant differences at 90 days after both RT and 45 °C storage. Therefore, storage at 4 °C preserved the particle size and capsaicin content in the NLC up to 90 days. The remaining capsaicin in the chili solution and NLC_Cs is shown in Figure 2a,b, respectively.

### 2.3. TEM Analysis

**Figure 3:**
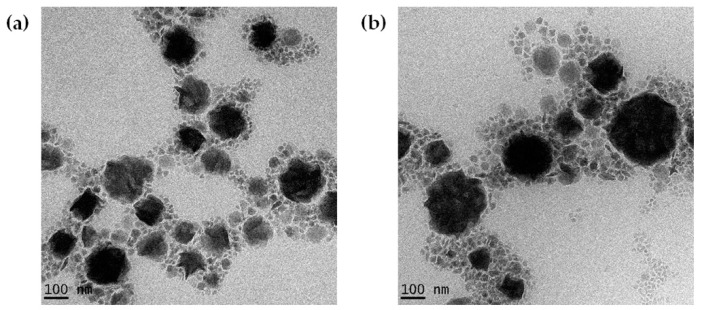
Transmission electron microscopy (TEM) images of nanostructured lipid carriers (NLC) prepared by high-shear homogenization (HSH) with high shear intensity; (**a**) blank NLC and (**b**) chili-extract-loaded NLC.

The NLCs prepared by HSH with high shear intensity were almost spherical and uniform in shape, with smooth surfaces. The particle diameters were approximately distributed between 100 and 200 nm. The chili-extract-loaded nanoparticles (NLC_C) were larger than the blank nanoparticles (NLC_B), implying the successful entrapment of the chili extract in the lipid-based nanoparticles. The morphologies of the NLC_Bs and NLC_Cs are shown in Figure 3a,b, respectively.

### 2.4. HPLC Method Validation

According to the International Conference on Harmonization of Technical Requirements for Registration Pharmaceuticals for Human use (ICH) guidelines [44] and Association of Analytical Communities (AOAC) guidelines [45], the HPLC analytical method for capsaicin analysis was validated in terms of specificity, accuracy, precision, linearity, limit of detection (LOD), and limit of quantitation (LOQ).

The overall accuracy and precision of the capsaicin analytical method was found to be consistent with acceptance criteria in the guidelines. Good linearity of calibration curves of capsaicin was observed over the concentration range of 0.25–100 µg/mL by visual inspection and by calculating the correlation coefficient (R^2^ of 0.9996 ± 0.0004). Specificity of the analytical method is as illustrated from the representative HPLC chromatograms of capsaicin standard, the chili-extract-loaded nanostructured lipid carriers (NLC_Cs), and the gel formulation containing NLC_C (Gel NLC_C) was demonstrated and other details were summarized in a Supplementary Materials.

### 2.5. Characterization and Stability Study of Chili-Extract-Loaded NLCs Incorporated in Gel Formulation

**Figure 4:**
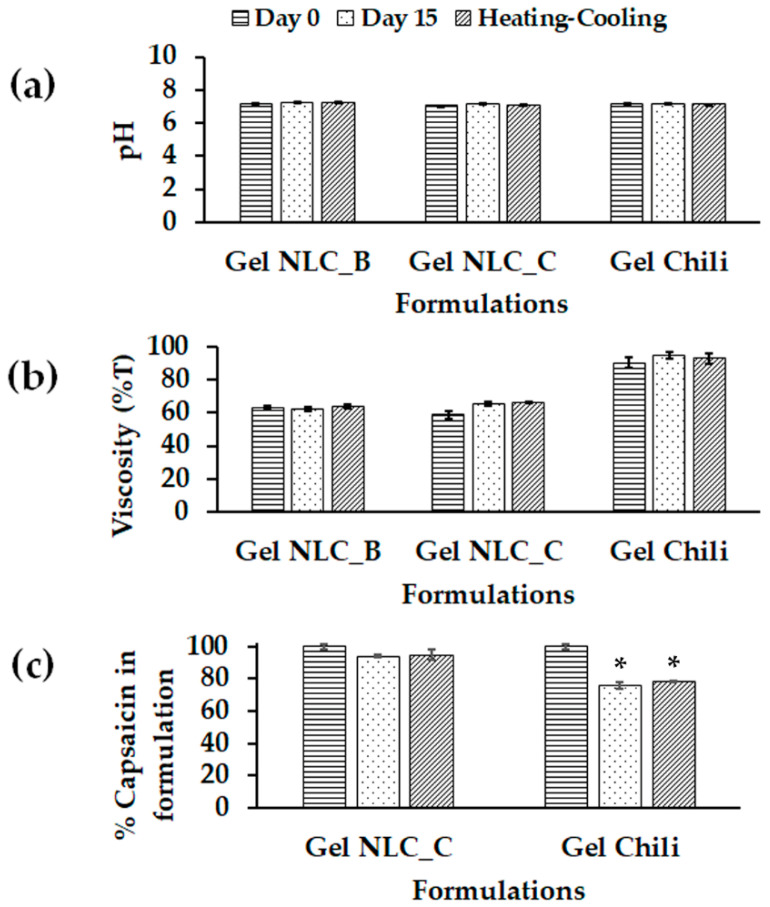
Stability study data of gel formulations: (**a**) pH, (**b**) viscosity, and (**c**) remaining capsaicin content in gel formulations after storage at room temperature and accelerated conditions. Abbreviations: NLC, nanostructured lipid carriers; Gel NLC_B, blank NLC-incorporate-in-gel; Gel NLC_C, chili-extract-loaded NLC-incorporate-in-gel; Gel Chili, chili-extract-incorporate-in-gel. * The values are significantly different (*p* < 0.05) compared to those for the initial formulation (Day 0).

The gel formulations of chili-extract-loaded NLCs and chili extract were stored in closed containers at room temperature for 15 days and under accelerated conditions by heating–cooling for seven cycles. The physical appearance, pH, viscosity, and capsaicin content were observed. The gel formulations were stable; the pH and viscosity of each formulation were not significantly different from those in the initial formulation (Day 0). The pH of all the gel formulations was approximately 7.0, which is within the acceptable limits for topical applications, 5.0–8.0 [46]. Moreover, chemical stability is one of the most important aspects in nanoparticle development [40,47]. The capsaicin content in the gel formulations after storage under two different conditions demonstrated the efficacy of NLCs in protecting capsaicin from oxidation and degradation during analysis or storage. A comparison of the capsaicin content between the chili-extract-incorporated-in-gel (Gel Chili) and NLC_C-incorporated-in-gel (Gel NLC_C) formulations revealed significant differences in Gel Chili from the initial formulation (Day 0). The stability of the gel formulation in terms of the pH, viscosity, and remaining capsaicin is shown in Figure 4a–c, respectively.

### 2.6. In Vitro Release Study

**Figure 5:**
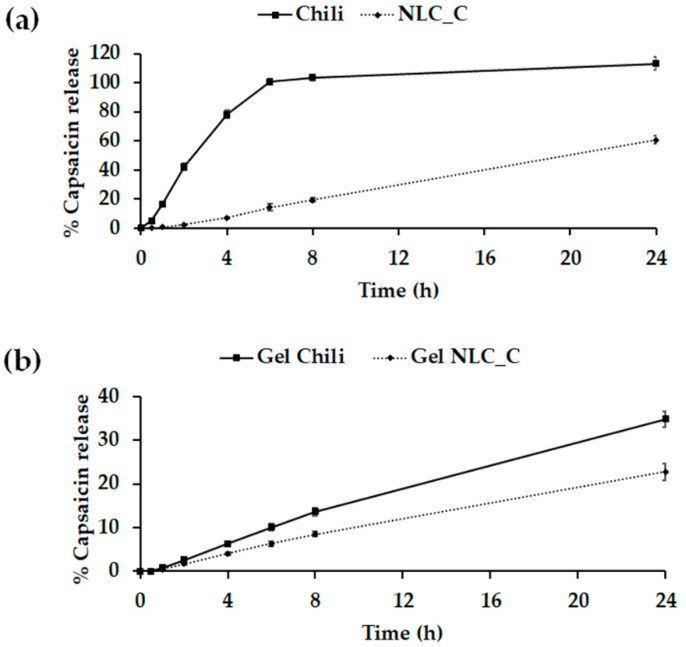
In vitro release study of (**a**) aqueous dispersions—chili extract and NLC_C—and (**b**) gel formulations; Gel Chili and Gel NLC_C, at various time points within 24 h. Abbreviations: NLC_C, chili-extract-loaded-nanostructured lipid carriers; Gel Chili, chili-extract-incorporate-in-gel; Gel NLC_C, chili-extract-loaded NLC-incorporate-in-gel.

The SnakeSkin^TM^ dialysis tubing is made from regenerated-cellulose, which is a commercially available membrane with a well-controlled molecular weight (MW) cut-off. The SnakeSkin^TM^ is widely used as a membrane model for an in vitro release study similar to a regular dialysis tube [48]. The release of NLC_Cs and Gel NLC_Cs was studied in vitro over 24 h using the dialysis method with PBS (pH 7.4)/ethanol (1:1) as the medium and compared with that of the chili extract solution and Gel Chili. Ethanol was added in the medium for increasing the solubility of capsaicin to reach the sink condition [49,50]. The release profiles of the NLC_C dispersion and Gel NLC_C showed a sustained release, which is attributable to the fact that capsaicin is a lipophilic compound. Thus, the capsaicin in chili prefers to stay within the lipid phase of NLCs rather than being released into an aqueous medium [51]. The release of capsaicin was slower from the gel formulation due to the release-retarding effect of the gelling agent’s polymeric matrix [5]. The sustained release may limit the side effects of the capsaicin [47]. The released profile was quantitatively determined using an HPLC system. The percentages of capsaicin release from NLC_C and Gel NLC_C are shown in Figure 5a,b, respectively. Moreover, Table 3 exhibits the correlation coefficient (R^2^) values of the kinetic release of capsaicin from different formulations. The release kinetics of capsaicin from NLC_C, Gel Chili, and Gel NLC_C were best fitted into the zero-order model. The zero-order kinetic model is related to various drug delivery systems, such as transdermal systems, slow release matrix tablets in coated forms, and osmotic systems [52]. The zero-order kinetic model is suitable for prolonged drug release because drug efficacy can be increased while dose frequency and toxicity can be reduced [53].

### 2.7. Study of NLC Distribution through the Skin Layers Using Fluorescence Microscopy

**Figure 6:**
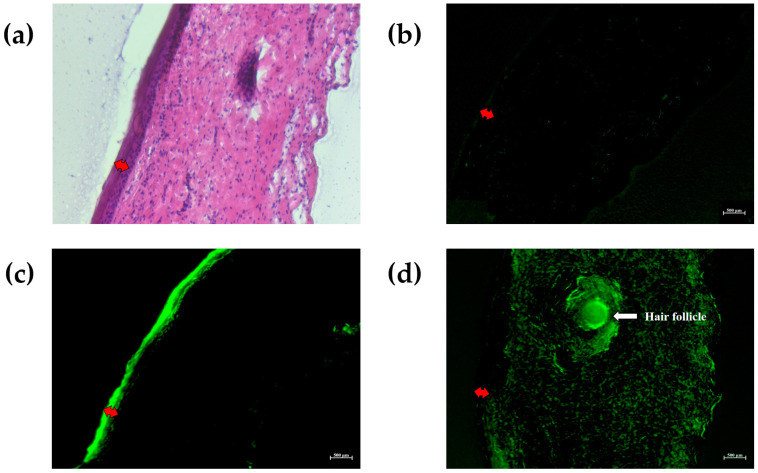
Fluorescence images of fluorescein distribution through the porcine skin samples; (**a**) a phase contrast image of the skin sample observed under a regular microscope; fluorescent images of the porcine skin sample treated with (**b**) PBS, pH 7.4; (**c**) fluorescein solution at 0.1% (*w*/*v*); and (**d**) NLC_F. Red arrows show the boundary of the epidermis layer. Abbreviations: NLC_F, fluorescein-loaded NLC.

Penetration images were obtained using various techniques such as optical microscopy, fluorescence microscopy, confocal laser scanning microscopy (CLSM), and Raman spectroscopy. Fluorescence microscopy is useful in cell physiology study, especially for studying skin delivery such as in permeation studies [54,55,56]. The porcine skin is a membrane barrier which can be defined by the skin layers like the human skin, enabling us to observe the fluorescent dye distribution and permeation in different layers [57]. Fluorescein is a hydrophobic fluorescent dye used as a marker for skin permeation studies. PBS (pH 7.4) was used as a negative control, and fluorescein solution (0.1% (*w/v*)) was used as a positive control. The ability of NLCs containing 0.1% (*w/v*) fluorescein (NLC_F) to permeate through porcine skin samples was studied for 24 h. The porcine skin samples were cut into vertically cross-sectional slices, and a fluorescent microscope was used to visualize the permeation profile through the skin layers in the fluorescein isothiocyanate (FITC) channel. A fluorescent image of the PBS-treated skin sample showed the skin auto-fluorescence. The image of the fluorescein solution (0.1% *w/v*) showed that most of the fluorescent dye permeated and accumulated in the stratum corneum layer. Moreover, the image of the nanodelivery system containing 0.1% (*w/v*) fluorescein showed that the fluorescent dye permeated deeply through the stratum corneum layer and was distributed in the dermis layer. The effective lipid-based nanoparticles were able to deliver the hydrophobic compound to the skin sample due to the occlusive effect, with the enhanced penetration of molecules through the stratum corneum, similar to observations from previous studies [36,58]. A phase contrast image of the same skin sample was observed under a regular microscope (under bright field) as shown in Figure 6a, and fluorescent images are shown in Figure 6b–d.

### 2.8. In Vivo Human Skin Irritation Test in Volunteers

**Figure 7:**
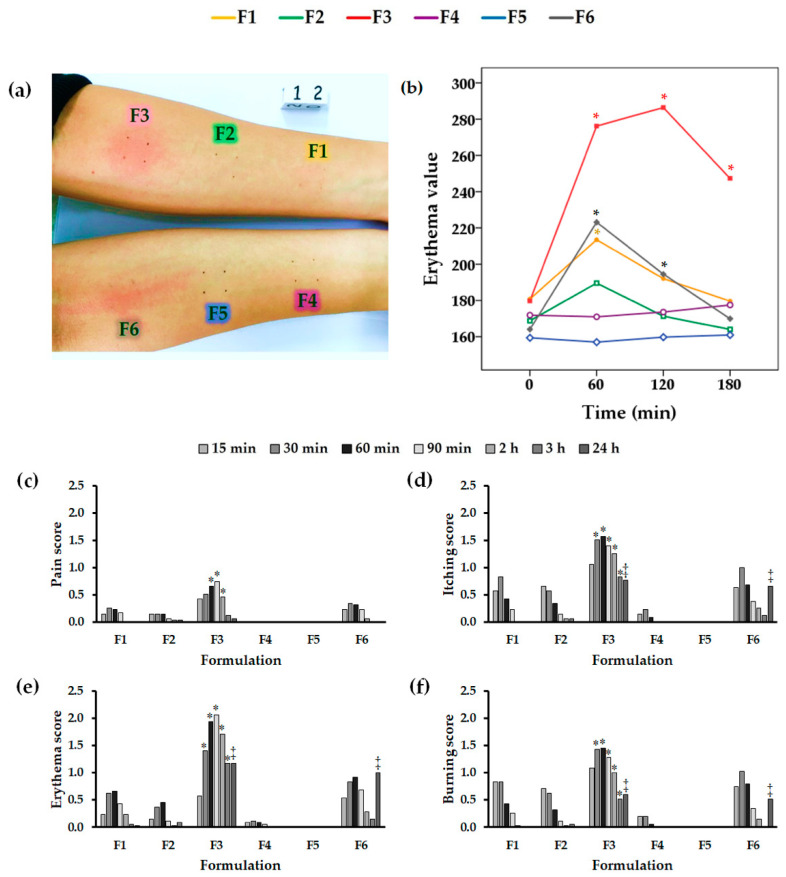
Erythema evaluation after applying each formulation on the human skin for 15 min and then removing it and leaving it on for 60, 120, and 180 min. (**a**) An example photo at 60 min; (**b**) erythema values determined with a Mexameter^®^; pain scores on a sensory scale at 15 min, 30 min, 60 min, 90 min, 2 h, 3 h, and 24 h, including for (**c**) pain, (**d**) itching, (**e**) erythema, and (**f**) burning. * The values are significantly different (*p* < 0.05) from the values for the other formulations, ‡ The values are significantly different (*p* < 0.05) from the values for the other formulations, except formulations F3 and F6. Note; F1: Gel NLC_C (chili-extract-loaded NLC-incorporated-in-gel formulation); F2: Gel NLC_B (blank NLC-incorporated-in-gel formulation); F3: Gel Chili (chili-extract-incorporated-in-gel formulation); F4: 5% sodium lauryl sulphate (SLS) solution; F5: Deionized water; F6: Commercial product.

Healthy male and female volunteers in the age group of 20–50 years were randomly included in this study. None of the volunteers had skin diseases or distinct signs of extrinsic skin aging in the test areas; however, irritation from capsaicin could occur in all age ranges. In the trial treatment, all sample types were applied to each individual who served as his/her own control. Erythema, a common sign of skin irritation, was evaluated using a Mexameter^®^, visual determination and sensory analysis. An example photograph illustrating irritation on a volunteer’s skin at 60 min is shown in Figure 7a. According to visual observation, Gel Chili (F3) and the commercial product (F6) induced more severe erythema than the other formulations. The erythema values measured by the Mexameter^®^ in Figure 7b demonstrates similar results. The erythema values from Gel Chili were significantly higher than those from the other formulations at 60, 120, and 180 min (*p* < 0.05), followed by those from the commercial product at 60 and 120 min (*p* < 0.05). On the contrary, only the erythema value for Gel NLC_C (F1) at 60 min was significantly higher than that at the initial time point and for the remaining samples (*p* < 0.05), whereas Gel NLC_B (F2) did not cause significant erythema at any time point, indicating the effectiveness of the NLCs in decreasing skin irritation. The graphical sensory data presented in Figure 7c–f express the feelings of the volunteers on rating scales—including pain, itching, redness, and burning—after applying the formulations. They correlate well with the erythema values. Gel Chili showed significantly higher ratings for skin irritation symptoms than the other formulations at 60 min to 2 h (*p* < 0.05) for pain, at 30 min to 3 h (*p* < 0.05) for itching and erythema (or redness), and 60 min to 3 h (*p* < 0.05) for burning sensation. Moreover, the ratings of itching, redness, and burning with Gel Chili at 24 h were significantly higher than those from others, except the commercial product. All the results highlight the advantage of lipid-based nanoparticles in reducing skin irritation by capsaicin. 

## 3. Materials and Methods

### 3.1. Materials

A capsaicin standard and a chili extract from *Capsicum chinense* were obtained from Bangkok Lab and Cosmetic Co., Ltd., Ratchaburi, Thailand. Glyceryl monostearate (GMS), cetyl alcohol (COH), and isopropyl myristate (IPM) were purchased from Namsiang, Bangkok, Thailand. Tween^®^ 80 and Span^®^ 80 were purchased from NOF Corporation, Tokyo, Japan. All the other chemicals were of the highest grade available. All the solutions were prepared with distilled water.

### 3.2. Preparation of Chili-Extract-Loaded Nanostructured-Lipid-Carrier (NLC) Dispersion

Chili-extract-loaded NLCs (NLC_C) containing 0.25% (*w*/*w*) capsaicin was prepared from lipid mixture and surfactant, consisted of glyceryl monostearate, cetyl alcohol, isopropyl myristate with appropriate HLB non-ionic surfactants. Suitable ratio of the selected liquid lipid, solid lipids surfactant mixtures, and the NLC preparation protocols were modified from the previous study [29]. Briefly, lipid phase and chili extract were melted at 70 °C under controlled stirring. An aqueous phase was mixed with a non-ionic surfactant and heated to 75 °C. Then, both phases were mixed together to form a pre-emulsion before subjecting to a particle-size-reduction step. Three different methods including (1) the combination of two processes, high-shear homogenization (HSH) followed by high-pressure homogenization (HPH); (2) HSH with a high shear intensity; and (3) HSH with a low shear intensity were performed for NLC preparation. In the first method, a lipid phase was gently added into an aqueous phase and thoroughly mixed using a high-shear homogenizer (Ultra-Turrax^®^ T25, IKA-Werke GmbH & Company KG, Staufen, Germany) at 5000 rpm for 5 min before a size-reducing step performed with a high-pressure homogenizer (APV1000, Invensys APV products, Silkeborg, Denmark) at 500 bar for 3 cycles. In the second, the emulsion mixture was mixed by using HSH with a high shear intensity at 10,000 rpm for 10 min, and in the third, HSH was used with a low shear intensity at 5000 rpm for 30 min. After the particle-size reduction step, all samples were left to cool to room temperature. The characteristics of the lipid nanoparticles derived from these three methods were compared to identify the most suitable technique for industrial-scale production. 

### 3.3. Preparation of Chili-Extract-Loaded NLCs Incorporated in Gel Formulation

The suitable method of chili-extract-loaded NLC preparation was applied in pilot-scale NLC production. The concentrated capsaicin at 0.25% (*w*/*w*) in the NLC suspension was further incorporated in a gel-based formulation to contain the final 0.075% (*w*/*w*) of capsaicin, a registered strength in the market. Gel-based formulation was a hydrophilic gel consisting of Carbomer and cellulose derivatives. The gel forming polymers were dispersed in distilled water containing propylene glycol (8% (*w*/*w*)). The NLC dispersion and gel-based formulation were mixed in a high-shear homogenizer 500 rpm for up to 10 min until homogeneous. 

### 3.4. HPLC Analysis

The precise capsaicin content in each formulation was determined by high-performance liquid chromatography (HPLC), using the HP1100 chromatographic system (Hewlett-Packard, Waldbronn, Germany) with a XBridge C18 (250 × 4.6 mm i.d., 5 µm, Waters, MA, USA). The mobile phase was a mixture of acetonitrile and 1% acetic acid at a ratio of 1:1 (*v*/*v*), and the flow rate was 1 mL/min at room temperature. The injection volume was 10 μL, and an ultra-violet (UV) detector was set at a wavelength of 280 nm [29]. The capsaicin analysis was validated in terms of accuracy, precision, and linearity. Capsaicin was quantified based on the calibration curve, which had good linearity in the range of 0.25–100 µg/mL. All samples were measured in triplicate.

### 3.5. Characterization of Chili-Extract-Loaded NLC Dispersion

#### 3.5.1. Particle-Size, Size-Distribution, and Zeta-Potential Analysis

The particle size, polydispersity index (PDI), and zeta potential (ZP) of the NLCs were determined using a dynamic light scattering (DLS), Zetasizer^®^ version 5.00 (Malvern Instruments, Worcestershire, UK). Samples were diluted with filtered deionized water (D.I. water) at 1:100 before measurement and kept at 25 °C. The experiments were performed in triplicate, and the results are expressed as the mean ± standard deviation (SD).

#### 3.5.2. Determination of Entrapment Efficiency (EE) 

The efficiency of the capsaicin entrapment in the NLC particles was determined by an indirect method. Briefly, chili extract and NLC_C colloidal suspension were placed in Amicon^®^ Ultra centrifugal filters (molecular weight cut-off (MWCO) 100 kDa; Merck Millipore) and centrifuged at 5000× *g* for 15 min at 4 °C (Sorvall^TM^ ST 16R, Thermo Fisher Scientific, Langenselbold, Germany). An aliquot of the filtrate part was dissolved in methanol at 1:20 ratio and further filtered to quantify the amount of free capsaicin using HPLC. The EE was calculated using Equation (1).

% EE = [(total amount of capsaicin − free capsaicin) × 100]/total amount of capsaicin(1)

#### 3.5.3. Transmission Electron Microscopy (TEM) Analysis 

The morphology of the chili-extract-loaded NLCs was determined by transmission electron microscopy (TEM, JEM-2010, JEOL, Tokyo, Japan). A single drop of diluted nanoparticles with D.I. water was dropped onto a copper grid and negatively stained with 1% (*w*/*v*) aqueous phosphotungstic acid solution. The copper grid was dried at room temperature prior to the analysis. The TEM investigation was performed at 100 kV.

### 3.6. Characterization of Gel Formulation

The gel formulations were characterized in terms of pH, viscosity, and capsaicin content. The pH was measured with a pH meter (inoLab^®^ pH Level 2, WTW GmbH, Weilheim, Germany), after the dilution of the formulation in D.I. water (1:10, *w*/*v*). The viscosity was determined with a rotary viscometer (RVDV-II+PCP, Brookfield Engineering Laboratories, Middleboro, MA, USA) using cone spindle CPE-42 at 25 ± 1 °C. The capsaicin content in the gel formulations was extracted with diethyl ether (1 h under mixing at room temperature) and was determined by the HPLC-UV method. All experiments were performed in triplicate, and the results are expressed as the mean ± SD.

### 3.7. Stability Study

All samples of the NLC_C dispersions prepared by the above-mentioned methods were kept in closed containers at room temperature for 30 days and evaluated for their stability. To ensure that the quality of the selected NLC_Cs were prepared by the suitable technique, the NLC_Cs were stored at different temperatures (4 °C, room temperature (RT), and 45 °C) for 90 days, and then, the stability was assessed in the terms of the particle size, uniformity (PDI), ZP, and remaining capsaicin content compared to that in the chili-extract solution. The chili-extract-loaded NLCs incorporated in the gel formulation (Gel NLC_C) and the chili-extract solution incorporated in a gel (Gel Chili) were stored at room temperature for 15 days and also in an accelerated condition (heating—cooling) for seven cycles. The NLC characteristics and the remaining capsaicin content were also evaluated after incorporation into the gel formulations. The remaining capsaicin content in each formulation was analyzed using the HPLC method as described in Section 3.4.

### 3.8. In Vitro Release Study 

The in vitro release behavior of the chili-extract-loaded NLCs and the chili-extract solution was studied using a modified Franz diffusion method [18,29]. An aliquot of freshly prepared colloidal suspension of chili-extract-loaded NLCs and chili-extract solution containing 0.25% (*w*/*w*) capsaicin was investigated through SnakeSkin^TM^ dialysis tubing (molecular weight cut-off 10 kDa; Thermo Fisher Scientific, Abbott Park, IL, USA), which is made from regenerated-cellulose. The SnakeSkin^TM^ dialysis tubing was soaked in a mixture medium containing phosphate buffer saline (PBS; pH 7.4) and absolute ethanol at a ratio of 1:1 (*v*/*v*) for 12 h before mounting in a Franz diffusion cell. Franz diffusion cells with a diffusion area of 2.00 ± 0.42 cm^2^ were used to study in vitro skin permeation. A receptor chamber (12 mL) was filled with the mixture medium and kept at 32 ± 2 °C with gentle stirring at 200 rpm. Samples (0.8 mL) from the receiving medium were drawn and immediately renewed with fresh medium at 0, 0.5, 1, 2, 4, 6, 8, and 24 h. For the in vitro release study, gel formulations containing 0.075% (*w*/*w*) capsaicin were applied to donor compartments. The capsaicin released in vitro from the NLC dispersion and gel formulations was quantified using an HPLC-UV system at 280 nm with an 80 µL injection volume. The experiments were carried out in triplicate. The release data were analyzed to determine the correlation coefficient (R^2^) and release kinetics of the NLC dispersion and gel formulations using various mathematical models [59]:

- Zero order model *Q* = *Q*_0_ + *kt*;
- First order model *Q* = *Q*_0_ × *e^kt^*;
- Higuchi model *Q* = *k* × *t*^0.5^;

Where *Q* is quantity of capsaicin release in time *t*, *Q*_0_ is the initial concentration of capsaicin, *k* is the rate constant. The model which exhibited the highest correlation coefficient (R^2^) value for the capsaicin release data was considered as the best fit. 

### 3.9. Study of NLC Distribution through the Skin Layers Using Fluorescence Microscopy

In order to evaluate the distribution of the developed NLCs in different skin layers, a fluorescent dye, fluorescein, was entrapped in the NLCs instead of the chili extract. The skin employed in the study was harvested from the dorsal skin of stillborn piglets. The dye distribution profile in the porcine skin was determined using Franz diffusion cells. The receiver medium was a mixture of PBS (pH 7.4) and ethanol (1:1, *v*/*v*). The studied samples (500 µL) were the fluorescein-loaded NLC suspension, a fluorescein solution containing 0.1% (*w*/*v*) fluorescein, and PBS (pH 7.4). They were added into separate donor chambers, and incubated at 32 ± 2 °C for 24 h; the skin samples were then cleaned to remove excess formulation and rinsed with 30 mL of D.I. water. Next, the skin samples were blotted with paper towels until dry. The porcine skin samples were frozen in the Tissue-Tek^®^ optimum cutting temperature (O.C.T.) compound with dry ice and sectioned to produce 5 µm-thick cross-sectional slices at −21 °C using a cryostat microtome (Leica CryoStat, CM3050S, Wetzlar, Germany). The permeation profile through the skin layers was visualized in the vertical sections with a fluorescent microscope (Zeiss Fluorescent microscopy with AxioCam 105 Color camera, Göttingen, Germany). Fluorescent images were captured from the slides using a Zeiss fluorescent microscope in the fluorescein isothiocyanate (FITC) channel, and the files were processed using Zen Pro 2.3. 

### 3.10. In Vivo Human Skin Irritation

The patch test was performed to evaluate the irritation of skin by the capsaicin formulations. The protocol was approved by Ethical Review Committee, Faculty of Pharmacy, Chiang Mai University (protocol number 13/2017). Thirty healthy volunteers, male and female between the ages of 20 and 50 years old, were enrolled in the study. Three 0.075% (*w*/*w*) capsaicin formulations—chili-extract-loaded NLCs incorporated in gel formulation (Gel NLC_C), chili extract incorporated in gel formulation (Gel Chili), and the commercial formulation along with positive (5% (*w*/*v*) sodium lauryl sulphate (SLS) solution) and negative (blank gel-based formulation (Gel NLC_B) and deionized water) controls—were applied on and evaluated in the same person across six different areas (1.5 × 1.5 cm) on clean forearms. Using a plastic spatula, 50 mg of each samples were applied and left in contact with the skin for 15 min before thorough removal with wipes. The skin redness (erythema) before the application and after the removal of the remaining products from the skin for 60, 120, and 180 min was evaluated by visual observation and using a Mexameter^®^ (CK Electronic, Cologne, Germany) [10,60]. The volunteers reported the sensory severity of skin irritation on a scale from 0 to 3 (0: no; 1: slight; 2: moderate; 3: severe) in terms of pain, itching, erythema, and burning [10]. Sensory data were recorded at 15, 30, 60, 90, 120, and 180 min and 24 h after the removal the formulations from the skin.

### 3.11. Statistical analysis

Data were collected from three independent experiments and are shown as mean ± SD. Statistical analysis was performed using *t*-tests, ANOVA, and Tukey’s multiple comparison test, using SPSS version 19.0 (IBM^®^ SPSS Statistics, Armonk, NY, USA). The statistical significance of differences less than 0.05 (*p*-values < 0.05) was considered statistically significant.

## 4. Conclusions

The techniques for preparing and scaling up chili-extract-loaded lipid-based nanocarriers can be performed by high-pressure homogenization and high-shear homogenization methods; however, in this study, a modified high-shear-homogenization approach with high shear intensity was proven as the most suitable for NLC production due to its product outcome, simplicity, versatility, and cost effectiveness for industry. Chili-extract-loaded NLCs containing a high capsaicin concentration (0.25%) revealed an optimal particle size around 200 nm, narrow size distribution, high entrapment efficiency, and good physicochemical stability. According to in vitro release and permeability studies, the chili-extract-loaded NLC formulation was more effective in terms of sustained release and distribution in the skin than the unloaded formulation. Furthermore, the gel-based formulation with the chili-extract-loaded NLCs was less irritating to human skin than the chili extract not loaded in NLCs and a commercial product. Therefore, the present study shows that chili-extract-loaded NLCs are suitable as a non-invasive drug delivery system for delivering capsaicin through the skin with minimal potential for irritation.

## Figures and Tables

**Table 1:** Stability study of NLCs prepared by different methods—the combination of high-shear homogenization (HSH) and high-pressure homogenization (HPH), and high-shear homogenization (HSH) with different shear intensities—following storage at room temperature for 30 days.

Characterization	Formulation	Day	HSH + HPH	HSH (High Intensity)	HSH (Low Intensity)
Size (nm)	NLC_B	0	70.70 ± 3.46	91.84 ± 3.15	119.89 ± 3.20
		7	83.12 ± 6.82	110.26 ± 3.26	127.75 ± 2.38
		14	87.53 ± 1.55	110.70 ± 3.46	126.35 ± 1.64
		30	95.30 ± 4.25	111.75 ± 4.05	184.30 ± 2.11 *
	NLC_C	0	90.70 ± 0.66	158.30 ± 2.39	170.49 ± 4.45
		7	94.81 ± 3.75	153.74 ± 3.23	174.43 ± 9.55
		14	98.10 ± 1.24	155.45 ± 4.84	178.80 ± 5.78
		30	157.60 ± 2.60 *	156.30 ± 6.88	222.80 ± 2.23 *
PDI	NLC_B	0	0.29 ± 0.02	0.21 ± 0.02	0.25 ± 0.06
		7	0.31 ± 0.02	0.26 ± 0.05	0.31 ± 0.13
		14	0.30 ± 0.05	0.25 ± 0.01	0.29 ± 0.05
		30	0.38 ± 0.03	0.26 ± 0.02	0.58 ± 0.06 *
	NLC_C	0	0.29 ± 0.03	0.25 ± 0.01	0.35 ± 0.01
		7	0.30 ± 0.10	0.26 ± 0.02	0.39 ± 0.01
		14	0.31 ± 0.04	0.24 ± 0.02	0.36 ± 0.03
		30	0.61 ± 0.03 *	0.28 ± 0.01	0.54 ± 0.03 *
ZP (mV)	NLC_B	0	−19.88 ± 1.41	−30.22 ± 1.02	−29.20 ± 0.61
		7	−16.08 ± 1.83	−31.30 ± 4.36	−27.20 ± 0.61
		14	−19.00 ± 0.66	−25.38 ± 0.83	−26.20 ± 0.72
		30	−18.78 ± 0.56	−29.39 ± 2.75	−14.35 ± 1.45 *
	NLC_C	0	−24.97 ± 0.91	−33.74 ± 0.71	−32.76 ± 0.60
		7	−27.64 ± 1.11	−38.10 ± 0.43	−30.76 ± 0.70
		14	−26.29 ± 0.48	−35.66 ± 0.98	−24.55 ± 0.53
		30	−17.29 ± 0.44 *	−39.27 ± 0.28	−14.33 ± 0.74 *

Abbreviations: PDI, polydispersity index; ZP, zeta potential; NLC_B, blank nanostructured lipid carriers; NLC_C, chili-extract-loaded nanostructured lipid carriers. * The values are significantly different (*p* < 0.05) compared to initial formulation (Day 0).

**Table 2:** Stability study of NLC_C formulation produced by HSH (high shear intensity for short time), following storage at different conditions for 90 days.

Day	Characterization	4 °C	Room Temperature	45 °C
0	Size (nm)	180.20 ± 1.33		
	PDI	0.22 ± 0.02		
	ZP (mV)	−35.84 ± 0.78		
30	Size (nm)	187.22 ± 9.65	181.20 ± 5.27	155.32 ± 2.27 *
	PDI	0.31 ± 0.03	0.25 ± 0.03	0.22 ± 0.02
	ZP (mV)	−34.72 ± 1.53	−34.46 ± 0.78	−34.16 ± 1.11
60	Size (nm)	198.30 ± 24.43	179.60 ± 3.55	160.84 ± 5.62 *
	PDI	0.39 ± 0.08	0.29 ± 0.04	0.26 ± 0.04
	ZP (mV)	−40.16 ± 0.44	−41.34 ± 0.39	−45.74 ± 1.72
90	Size (nm)	196.58 ± 17.46	180.72 ± 5.36	205.98 ± 3.10 *
	PDI	0.49 ± 0.05	0.30 ± 0.04	0.30 ± 0.02
	ZP (mV)	−41.50 ± 1.93	−45.56 ± 1.52	−43.34 ± 1.24

Abbreviations: PDI, polydispersity index; ZP, zeta potential. * The values are significantly different (*p* < 0.05) compared to those for the initial formulation (Day 0).

**Table 3:** Correlation coefficient (R^2^) values of the capsaicin-release kinetic models.

Formulation	Zero-Order	First-Order	Higuchi
Chili extract	0.5883	0.3872	0.8007
NLC_C	0.9988	0.5554	0.9334
Gel Chili	0.9940	0.6829	0.9714
Gel NLC_C	0.9957	0.6656	0.9694

Abbreviations: NLC_C, chili-extract-loaded-nanostructured lipid carriers; Gel Chili, chili-extract-incorporate-in-gel; Gel NLC_C, chili-extract-loaded NLC-incorporate-in-gel.

## Supplementary Materials

The following are available online, Figure S1: Representative HPLC chromatograms of capsaicin formulations and Table S1: Intraday and interday accuracy and precision results for capsaicin.
