# Supplementary material for: Nanomaterial Lipid-Based Carrier for Non-Invasive Capsaicin Delivery; Manufacturing Scale-Up and Human Irritation Assessment
Source: Molecules. 2020 Nov 27;25(23):5575. doi: 10.3390/molecules25235575 (PMC7729988; doi:10.3390/molecules25235575)
Supplement: Supplementary file 1 [file molecules-25-05575-s001.pdf]

HPLC chromatograms of NLC dispersion and gel formulation

The HPLC chromatograms of 100 μg/mL capsaicin standard solution, 0.25% (*w*/*w*) capsaicin in chili-extract-loaded nanostructured lipid carriers (NLC_Cs), 0.075% (*w*/*w*) capsaicin in NLC_C-incorporated-in-gel (Gel NLC_C) and gel-based formulation without capsaicin are shown in Figure 1. All formulations were dissolved in methanol. The HPLC chromatogram of chili extract consisting of capsaicin and dihydrocapsaicin revealed the retention times of 10.00 and 14.00 min.

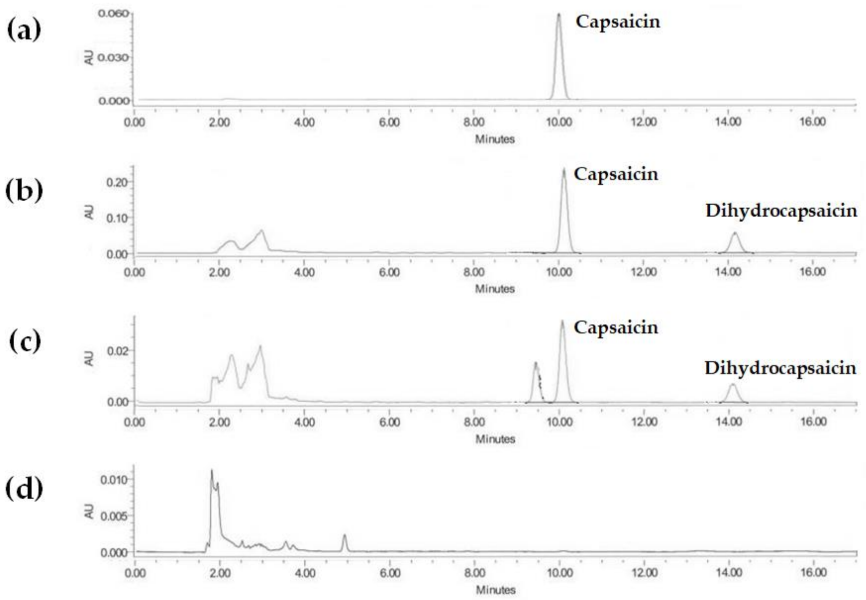

Figure 1. Representative HPLC chromatograms of (a) capsaicin standard solution, (b) chili-extract-loaded nanostructured lipid carriers (NLC_Cs), (c) NLC_C-incorporated- in-gel (Gel NLC_C) and (d) gel-based formulation.

HPLC Method Validation

A simple and validated RP-HPLC method for capsaicin according to the ICH and AOAC guidelines demonstrated the good specificity, linearity, accuracy, and precision in the range of 0.25–100 µg/mL. The limit of detection (LOD) and limit of quantitation (LOQ) were 0.1 and 0.25 µg/ml. The results of accuracy and precision are summarized in Table 1. The method was found to be suitable for the analysis of capsaicin from the entrapment, content, and release studies.

Table 1. Intraday and interday accuracy and precision results for capsaicin. Results are expressed as mean ± SD.

	Intraday (n=3)			Interday (n=9)		
Capsicin concentration (µg/ml)	Measured concentration (µg/ml)	Recovery (%)	RSD (%)	Measured concentration (µg/ml)	Recovery (%)	RSD (%)
0.25	0.26 ± 0.03	103.12 ± 2.52	1.60	0.26 ± 0.02	103.18 ± 8.04	1.44 ± 0.29
0.5	0.54 ± 0.03	108.56 ± 6.37	0.88	0.54 ± 0.04	107.24 ± 8.90	0.81 ± 0.12
1	1.09 ± 0.02	109.21 ± 2.37	0.22	1.07 ± 0.09	107.27 ± 9.16	0.24 ± 0.03
2.5	2.53 ± 0.01	101.36 ± 0.25	0.09	2.48 ± 0.06	99.00 ± 2.51	0.24 ± 0.27
5	5.10 ± 0.02	101.91 ± 0.30	0.45	4.98 ± 0.12	99.63 ± 2.45	0.50 ± 0.08
10	9.88 ± 0.01	98.76 ± 0.10	0.10	9.77 ± 0.27	97.74 ± 2.75	0.33 ± 0.22
25	24.51 ± 0.03	98.05 ± 0.13	0.19	25.07 ± 0.51	100.30 ± 2.03	0.59 ± 0.35
50	49.99 ± 0.09	99.99 ± 0.19	0.20	50.07 ± 0.13	100.13 ± 0.27	0.49 ± 0.41
100	100.13 ± 0.04	100.13 ± 0.04	0.09	99.97 ± 0.15	99.97 ± 0.15	0.46 ± 0.44

Abbreviations: RSD, relative standard deviation.

Participant Information Sheet

1. **Research Project Title**

   Chili Extract Delivery System for Minimizing Skin Irritation

2. **Invitation**

   You are being invited to take part in this research project. Before you decide to participate in this project, it is important to understand why the research is being done and what it will involve. Please take time to read the following information carefully and discuss it with others if you wish. Ask us if there is anything that is unclear or if you want more information. Take time to decide whether or not you wish to take part. Thank you for reading this.

3. **What is this trial about?**

   Chili extract has been incorporated in many topical products applied for various diseases such as musculoskeletal pain, osteoarthritis, rheumatoid arthritis, diabetic neuropathy, and post-herpetic neuralgia. However, the major problem of chili extract after its application can cause on skin irritation. Therefore, the development of chili-extract delivery system has been performed in order to harness the benefits of capsaicin while avoiding its irritation effects.

4. **What is the project's purpose?**

   1. To study the skin irritation effects from the chili-extract delivery system incorporated in a topical formulation.
   2. To evaluate the sensory severity of skin irritation and satisfaction in the chili-extract delivery system incorporated in a topical formulation.

5. **Why have I been chosen?**

   You have been chosen because you are a healthy volunteer with normal skin, so called a healthy volunteer, who is appropriate to be a volunteer for skin irritation study for the developed topical formulation.

6. **Do I have to take part?**

   It is up to you to decide whether or not to take part in this study. Your decision to participate is voluntary.

   If you do decide to take part, you will be asked to sign a consent form. You will be given a copy of this signed consent form for your record. Additionally, you are still free to withdraw at any time and without giving a reason.

7. **What will happen to me if I take part?**

Before you start the trial, the doctor or staff will talk to you about the trial. If you agree to participate in the trial, you will have to sign an informed consent.

Then, your forearms will be screened for the existing open wound and skin disease. At least one week before the study, you need to avoid using topical steroid products or other topical medicines.

During a visit day, the staff will show you how to apply the formulation on your trial area. Your arms will be washed and marked the trial area in six areas (1.5 × 1.5 cm per area) before you apply the formulations. The formulations will be left in contact with the skin for 15 min, and then they will be removed by wipes. Patch test will be performed on your forearms and the erythema will be evaluated by using Mexameter® (CK Electronic, Cologne, Germany). The skin irritation will be visual analyzed by capture and followed by skin-color measurement with probe analysis (Mexameter®) at various times; before and 60, 120, and 180 min after exposure to the formulations and controls. Self-observations in a questionnaire about your symptoms, and satisfaction on these formulations will be observed at each time; 15, 30, 60, 90, 120, 180 min and 24 h after applied the formulations. It will take approximately 5 minutes to complete the questionnaire.

8. **What are the possible side-effects of taking part?**

The unwanted side effect after applying the chili extract formulations is skin irritation including pain, itching, erythema and burning; however, the severe skin irritation is a rare case which upon an individual response. If you suffer these or any other symptoms and become worried, please contact the doctor or the staff.

9. **What will happen if I don't want to carry on with the trial?**

If you decide not to take part or withdraw from the trial, this will not affect the standard of care you receive or loss of any benefits you would otherwise receive. If you want to stop your participation in the trial, please tell the doctor or staff.

10. **What are possible benefits of taking part?**

We cannot guarantee that you will receive any benefits from this study; however, results may benefit patients who have used the developed topical formulation from chili-extract delivery system for pain relief in the future.

11. **What if something goes wrong?**

If you suffer or become severe skin irritation as a result of participating in this trial, necessary medical treatment will be made available at no cost to you to the full extent provided.

12. **What will happen to information about me?**

    By signing the consented form, you consent to doctor and relevant staff for collecting and using your personal information in the trial. Any information obtained in this study that can identify you will remain confidential. The information collected in this trial will be identified by a code number. Only doctor and the study team will be able to link the code number to you personally. Your information will only be used for the purpose of this trial and it will only be disclosed with your permission.

13. **Who has ethically reviewed the project?**

    This project has been ethically approved by Ethical Review Committee, Faculty of Pharmacy, Chiang Mai University (protocol number 13/2017).

14. **Contacts for further information**

    - Phunsuk Anantaworasakul, Department of Pharmaceutical Sciences, Faculty of Pharmacy, Chiang Mai University, Chiang Mai, Thailand. Tel: +6686 919 1791, email: phunsuk@gmail.com
    - Assist. Prof. Dr Chadarat Amprasavate, Department of Pharmaceutical Sciences, Faculty of Pharmacy, Chiang Mai University, Chiang Mai, Thailand. Tel: +6653 944 342, email: chadarat.a@cmu.ac.th
    - Assoc. Prof. Dr Songyot Anuchapreeda, Division of Clinical Microscopy, Department of Medical Technology, Faculty of Associated Medical Sciences, Chiang Mai University, Chiang Mai, Thailand. Tel: +6653 949 237, email: songyot.anuch@cmu.ac.th
    - Assist. Prof. Napatra Tovanabutra (MD), Division of Dermatology, Department of Internal Medicine, Faculty of Medicine, Chiang Mai University, Chiang Mai, Thailand. Tel: +6653 935 482, email: napatra.to@cmu.ac.th

15. Flow chart

The doctor or staffs will talk to you about the trial. If you agree to participate in the trial, you will have to sign informed consent.

Your arms will be washed and marked the trial area in six areas (1.5 × 1.5 cm per area) before you apply the formulations. Patch test will be performed on the forearms of volunteers and the erythema will be evaluated by using Mexameter®

The 50 mg of each formulations will be left in contact with the skin for 15 min, and then will be removed by wipes.

The skin irritation will be measured before and 60, 120, and 180 min after exposure to the formulations and controls. At each time point, a visual analysis was captured and followed by probe analysis (Mexameter®).

You will further fill in some questionnaires about your symptoms and satisfaction of formulation on a scale at each time points; 15, 30, 60, 90, 120, 180 min and 24 h after application.

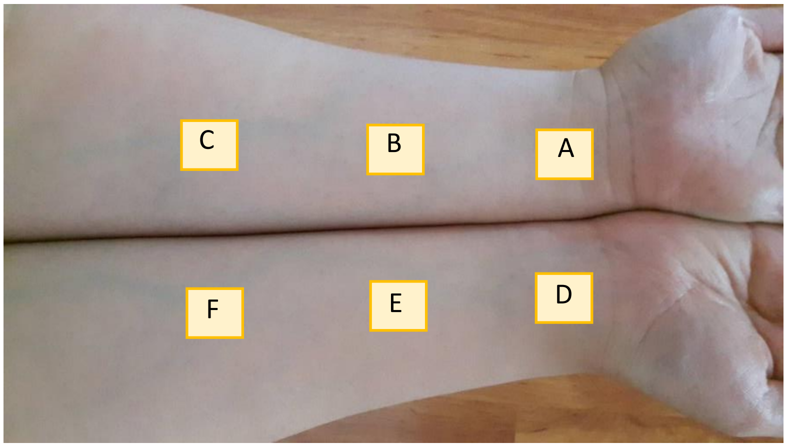

The six-test areas for samples application
